# Prevalence and awareness of hepatitis B and hepatitis C and vaccine-induced immunity to hepatitis B: Findings from the Canadian Health Measure Survey, 2016–2019

**DOI:** 10.14745/ccdr.v51i67a03

**Published:** 2025-07-01

**Authors:** Simone Périnet, Anson Williams, Qiuying Yang, Laurence Campeau, Jacqueline Day, Lindsey Lamboo, Emma R Lee, Carla Osiowy, Nashira Popovic

**Affiliations:** 1 Centre for Communicable Diseases and Infection Control, Public Health Agency of Canada, Ottawa, ON; 2 National Microbiology Laboratory, Public Health Agency of Canada, Winnipeg, MB; 3 Department of Medical Microbiology and Infectious Diseases, University of Manitoba, Winnipeg, MB

**Keywords:** hepatitis B, hepatitis C, viral hepatitis, estimates, awareness, prevalence, Canada

## Abstract

**Background:**

Hepatitis B virus (HBV) and hepatitis C virus (HCV) infections are sexually transmitted and blood-borne infections that Canada is committed to eliminate as public health concerns. Accurate epidemiological estimates require cross-sectional data as input. The objective of this study was to estimate the prevalence of present HBV infection (hepatitis B surface antigen-positive) and proportion aware of their infection, the vaccine-induced HBV immunity, the prevalence of HCV antibodies (anti-HCV-positive), the prevalence of present HCV infection (RNA-positive) and proportion aware of their infection, in the household population in Canada. These outcomes were also examined by selected demographic characteristics.

**Methods:**

A total of 7,543 sera from participants of the Canadian Health Measure Survey (CHMS) cycles 5 (2016–2017) and 6 (2018–2019) who consented to participate in Statistics Canada’s Biobank were tested to determine their HBV and HCV status. Information from the CHMS household questionnaire was linked to the laboratory results to report on sociodemographic characteristics and awareness of infection.

**Results:**

The stored serum combined response rate for this study, which takes into account households’ and respondents’ participation in the CHMS and the Biobank was 42.8%. The estimated prevalence of present HBV infection among people aged 14 to 79 years was 0.4% (95% CI: 0.1%–0.7%), of whom 49.0% (95% CI: 15.4%–82.6%) were aware of their infection. An estimated 39.0% (95% CI: 37.0%–41.0%) of people aged 11 to 79 years had laboratory evidence of vaccine-induced HBV immunity. An estimated 0.5% (95% CI: 0.2%–0.8%) of people aged 14 to 79 years were positive for anti-HCV, and 0.2% (95% CI: 0.0%–0.3%) had a present infection (RNA-positive), of whom 51.2% (95% CI: 9.5%–92.9%) were aware of their infection.

**Conclusion:**

Cross-sectional data using nationally representative surveys are essential in assessing the burden of viral hepatitis.

## Introduction

Canada is committed to eliminating hepatitis B virus (HBV) and hepatitis C virus (HCV) infections as public health concerns ((1)). Certain populations and communities are disproportionately affected by these sexually transmitted and blood-borne infections (STBBI) ((2,3)). In Canada, people from countries where HBV is common have the highest estimated HBV prevalence ((3)), and people who inject drugs (PWID) have the highest HCV prevalence. However, people born between 1945 and 1975 account for the largest absolute number of people living with HCV ((3)), due to the large demographic weight of this group. Most infections in this cohort result from medical procedures or past injection drug use ((4)).

Cross-sectional data from representative surveys are essential to estimate prevalence and awareness, which is the first step in tracking Canada’s progress towards eliminating viral hepatitis as a public health concern. The Canadian Health Measure Survey (CHMS), conducted by Statistics Canada, collects self-reported health information and direct physical measures, including blood samples. Specimens are stored as part of the Statistics Canada Biobank for future studies. The last viral hepatitis estimates derived from the CHMS were for the period 2007–2009 and 2009–2011 ((5)). In this study, Statistics Canada Biobank blood samples from the 2016–2017 and 2018–2019 CHMS cycles were used to estimate the prevalence and proportion aware for HBV and HCV infections among people aged 14 to 79 years living in Canadian households for the 2016–2019 period, by sociodemographic characteristics. Vaccine-induced HBV immunity among people aged 11 to 79 years and children aged 11 to 17 years for the same period is also reported.

## Methods

### Study design

The CHMS has been described in detail elsewhere ((6)). It collects health information through a questionnaire, direct physical measurement, and blood and urine samples to test for chronic and infectious diseases. The CHMS is designed to be representative of Canada’s provincial population (excluding the territories). The sampling frame captured 97% of the household population for the cycles used ((7)), but excludes Indigenous people living in communities, full-time members of the Canadian Forces, the institutionalized population, and residents of some remote regions. The Public Health Agency of Canada (PHAC) partnered with Statistics Canada and the National Microbiology Laboratory (NML) to conduct HBV and HCV testing on a subsample of serum specimens that were stored as part of the Statistics Canada Biobank for cycles 5 (2016–2017) and 6 (2018–2019) of the CHMS. Informed consent from respondents to have their samples stored as part of the Biobank was obtained at the time of participation, and all respondents who provided biological samples have consented to the mandatory reporting of reportable disease to provincial authorities. All participants who had present HBV or HCV infection were contacted by Statistics Canada through multiple phone attempts to confirm their mailing address. When contact was made, and mailing address confirmed, a results report was sent to the participant via registered mail. The project was approved by the Health Canada and PHAC Research Ethics Board (protocol number REB 2021–001P).

### Specimen collection

Blood samples were collected from consenting respondents, allowed to clot at room temperature, protected from light for 30 minutes, and centrifuged at 8°C for 15 minutes at 3,400 RPM. Each serum was aliquoted and stored in laboratory freezers until weekly shipment to the Statistics Canada biorepository, where they were stored at −80°C until sent to the NML reference laboratory for analysis.

### Laboratory methods

Respondent serum samples were analyzed for the qualitative detection of HBV markers using the electrochemiluminescence immunoassay (ECLIA) technique on the Cobas e411 Immunoanalyzer (Roche Diagnostics, Laval, Québec). All samples underwent testing for HBV surface antibodies (anti-HBs) and total HBV core antibodies (anti-HBc) using the Elecsys Anti-HBs II Assay and the Elecsys Anti-HBc II, respectively. Samples that screened positive for anti-HBc were tested for the HBV surface antigen (HBsAg) using the Elecsys HBsAg II Assay on the same platform. Initial HBsAg positive results were confirmed using the Elecsys HBsAg Confirmatory Test.

The qualitative detection of HCV antibodies (anti-HCV) was performed using ECLIA on the Cobas e411 Immunoanalyzer (Roche Diagnostics, Laval, Québec) and a chemiluminescent microparticle immunoassay (CMIA) using the Architect i2000 SR Immunoanalyzer (Abbott Diagnostics, Mississauga, Ontario). If the initial Elecsys Anti-HCV II Assay was positive, the ARCHITECT Anti-HCV Assay was used as a confirmatory test. Anti-HCV-positive samples were assessed qualitatively for detection of RNA using reverse transcriptase polymerase chain reaction (RT-PCR) with the Xpert HCV Viral Load Assay using the GeneXpert system (Cepheid Canada, Markham, Ontario).

Each sample batch included positive and negative controls.

### Statistical analysis

All statistical analyses were carried out using SAS Enterprise Guide 7.1 and were weighted to account for survey design and non-response. Weighted estimates are representative of the population covered by the survey, in this case the household population in provinces of Canada. Ninety-five percent confidence intervals (95% CI) were computed using the Bootstrap method with 500 repetitions. For national-level estimates, 22 degrees of freedom were applied, and for regional-level estimates, 2–10 degrees of freedom were used, following the CHMS user guide (available upon request to Statistics Canada). Second order (Satterthwaite) likelihood ratio chi-square tests were conducted to assess statistical significance at a level of 5%. Due to variations in the available degrees of freedom, regional-level estimates were assessed using 95% CIs rather than producing *p*-values. Data were not shown for cells with five or less observations and absolute number estimates and proportions were rounded to the nearest hundred units and to one decimal, respectively.

### Variable definitions

The detection of HBsAg was a marker of present HBV infection. An anti-HBs result was considered positive if ≥10 IU/L. Positive anti-HBs in combination with negative anti-HBc was used as an indication of vaccine-induced HBV immunity. Two respondents with unclear anti-HBs/anti-HBc status were excluded from the immunity analysis. Anti-HCV was used as a marker of past or present HCV infection, and HCV RNA as a marker of present infection (**Table 1**).

**Table 1:** Defining hepatitis B virus and hepatitis C virus infections and vaccine-induced immunity to hepatitis B

Characterization	Biomarkers
**HBV**	
Present infection	Hepatitis B virus surface antigen (HBsAg) positive
Vaccine-induced immunity	Hepatitis B virus core antibody (anti-HBc) negative and hepatitis B virus surface antibody positive^a^ (anti-HBs)
**HCV**	
Present or previous infection	Hepatitis C virus antibody (anti-HCV) positive
Present infection	Hepatitis C virus RNA positive

Abbreviations: anti-HBc, hepatitis B virus core antibody; anti-HBs, hepatitis B virus surface antibody positive; HBsAg, hepatitis B virus surface antigen; HBV, hepatitis B virus; HCV, hepatitis C virus

^a^ An anti-HBs result was considered positive if ≥10 IU/L

Participants who tested positive for HBsAg and indicated having hepatitis B were considered aware of their infection. These participants responded “hepatitis B” when asked 1) “What kind of liver disease or gallbladder problem do you have?”, or 2) “What type of hepatitis do you have?”, or 3) “Which sexually transmitted disease(s) or infection(s) have you been diagnosed with?”. Details on skip logic are available in the Statistics Canada data dictionaries.

Participants who tested positive for HCV RNA and indicated having hepatitis C were considered aware of their present infection. These participants responded “hepatitis C” when asked 1) “What kind of liver disease or gallbladder problem do you have?”, or 2) “What type of hepatitis do you have?”. Details on skip logic are available in the Statistics Canada data dictionaries.

Infection status for HBV and HCV, awareness of infection, and vaccine-induced HBV immunity were examined by sex, age group/birth cohorts, province or region, being born outside of Canada, being born in an intermediate-to-high prevalence country, history of injection drug use, household income, and highest education level. Age groups/birth cohorts were grouped in larger categories as needed for statistical power considerations. Countries of birth with a country- or region-specific anti-HCV or pooled HBsAg estimate of 2% or greater were classified as intermediate-to-high prevalence countries ((8–10)). Participants in the CHMS were asked whether they had ever injected or been injected with drugs for non-medicinal purposes. Household income in dollars was available for cycle 6, but only as an ordinal variable for cycle 5; the cycle 6 incomes were first grouped as deciles and the data was dichotomized as median or less (i.e., lower), and higher than median (i.e., higher). Categories of highest education level achieved were dichotomized as needed for statistical power considerations.

## Results

### Survey participants

The household response rate (i.e., the proportion of respondent households among households within the scope of the survey) for this study was 74.0%. The stored serum combined response rate (SerCRR) for this study was 42.8%. The SerCCR takes into account the household response rate, the number of respondents for each household, their participation in the questionnaire and physical examination, successful blood draw, and whether the serum fraction was stored. SerCRRs ranged from 40.5% (12–19 years) to 45.5% (40–59 years) across age groups.

Sera from 7,543 participants were included in the analysis. Among those participants, 499 were aged 11 to 13 years, 880 were aged 14 to 17 years, and 6,164 were aged 18 to 79 years (50% female; median [IQR] age, 40 [36] years) (**Table 2**).

**Table 2:** Description of the analyzed sample (n=7,543)

Characteristic	Unweighted frequency	Weighted (%)
**Sex**		
Female	3,712	50.2
Male	3,831	49.8
**Age groups**		
11–13	499	2.4
14–17	880	5.2
18–24	580	9.8
25–34	924	16.7
35–44	1,568	16.5
45–54	913	17.2
55–64	983	17.2
≥65	1,196	14.9
**Province or region of residence**		
Atlantic	924	6.5
Québec	1,842	22.8
Ontario	2,500	39.4
Prairies	1,234	18.0
British Columbia	1,043	13.2
**Born outside of Canada**		
Yes	1,937	32.1
No	5,605	67.9
Not stated	-	-
**Born in an intermediate-to-high HBV prevalence country^a^**		
Yes	1,206	20.4
No	6,318	79.5
Not stated	19	0.1
**Born in an intermediate-to-high HCV prevalence country^b^**		
Yes	279	4.4
No	7,245	95.5
Not stated	19	0.1
**Household income**		
Lower	3,881	51.1
Higher	3,662	48.9
**Education**		
Less than secondary school graduation	532	8.1
Secondary school graduation	1,295	20.7
Post-secondary graduation	4,294	63.0
Not stated or not applicable (respondent aged <18 years)	1,422	8.5
**Lifetime history of injection drug use**		
Yes	69	1.1
No	7,421	98.1
Not stated	53	0.8
Total	7,543	100.0

Abbreviations: HBV, hepatitis B virus; HCV, hepatitis C virus; -, cell size does not meet diffusion guidelines

^a^ Countries with estimated hepatitis B surface antigen (HBsAg) prevalence greater or equal to 2%

^b^ Countries with estimated hepatitis C antibodies prevalence greater or equal to 2%

### Hepatitis B prevalence and awareness

The estimated prevalence of present HBV infection in the household population aged 14 to 79 years was 0.4% (95% CI: 0.1%–0.7%). The prevalence among people born outside of Canada (1.2%; 95% CI: 0.5%–1.9%) was more than 10 times higher than the prevalence of those born in Canada (0.1%; 95% CI: 0.0%–0.1%; *p*=0.004). The prevalence among those born in intermediate-to-high prevalence countries was estimated at 1.9% (95% CI: 0.8%–3.1%) (**Table 3**). Among people with present HBV infection, 49.0% (95% CI: 15.4%–82.6%) were aware of their infection (**Table 4**).

**Table 3:** Estimated prevalence of present hepatitis B infection by selected characteristics, household population aged 14–79, Canada, 2016–2019 (n=7,044)

Characteristic	Weighted proportion (%)	95% CI	Weighted frequency (n)	95% CI	*p*
Overall	0.4	0.1–0.7	128,000	40,900–215,200	N/A
**Sex**					
Female	0.4	0.00–0.9	61,500	0–126,400	0.9
Male	0.5	0.1–0.8	66,600	10,500–122,700	
**Age groups**					
14–49	0.5	0.1–0.8	79,500	14,200–144,900	0.7
>50	0.4	0.1–0.7	48,500	6,900–90,200	
**Lifetime history of injection drug use**					
Yes	-				N/A
No	0.4	0.1–0.8	128,000	40,900–215,200	
**Born outside of Canada**					
Yes	1.2	0.5–1.9	116,700	35,600–197,900	0.004
No	0.1	0.0–0.1	11,300	276–22,400	
**Born in an intermediate-to-high HBV prevalence country^a^**					
Yes	1.9	0.8–3.1	116,700	35,600–197,900	0.005
No	0.05	0–0.1	11,300	300–22,400	
**Household income**					
Lower	0.4	0.1–0.7	58,500	12,900–104,000	0.7
Higher	0.5	0.0–1.0	69,600	3,000–136,200	
**Education^b^**					
Secondary school graduation or less	0.3	0.0–0.7	27,600	0–59,200	0.4
Post-secondary graduation	0.5	0.1–1.0	100,300	16,600–184,000	

Abbreviations: CI, confidence interval; HBV, hepatitis B virus; N/A, not applicable; -, cell size does not meet diffusion guidelines

^a^ Countries with estimated hepatitis B surface antigen (HBsAg) prevalence greater or equal to 2%

^b^ Among people aged >18 years (n=6,121)

**Table 4:** Awareness of present hepatitis B virus infection by selected characteristics, household population aged 14–79, Canada, 2016–2019 (n=32)

Characteristic	Weighted proportion (%)	95% CI	Weighted frequency (n)	95% CI	*p*
Overall	49.0	15.4–82.6	62,700	1,900–123,510	N/A
**Sex**					
Female	-				0.8
Male	53.1	19.5–86.8	35,400	1,600–69,200	
**Age groups**					
14–49	57.0	8.4–100.0	45,400	0–103,400	0.5
>50	35.7	0.0–80.4	17,300	0–36,100	
**Born outside of Canada**					
Yes	48.3	8.7–88.0	56,400	0–117,500	0.9
No	-				
**Born in an intermediate-to-high HBV prevalence country^a^**					
Yes	48.3	8.6–88.0	56,400	0–117,500	0.9
No	-				
**Household income**					
Lower	30.2	0.0–70.6	17,700	0–42,900	0.2
Higher	64.7	21.5–100.0	45,000	0–102,000	
**Education^b^**					
Secondary school graduation or less	55.2	17.2–93.2	44,900	0–99,500	0.4
Post-secondary graduation	-	-	-	-	

**Table:** 

Abbreviations: CI, confidence interval; HBV, hepatitis B virus; N/A, not applicable; -, cell size does not meet diffusion guidelines
^a^ Countries with estimated hepatitis B surface antigen (HBsAg) prevalence greater or equal to 2%
^b^ Among people aged >18 years (n=12)

### Vaccine-induced immunity to hepatitis B

**Figure 1:**
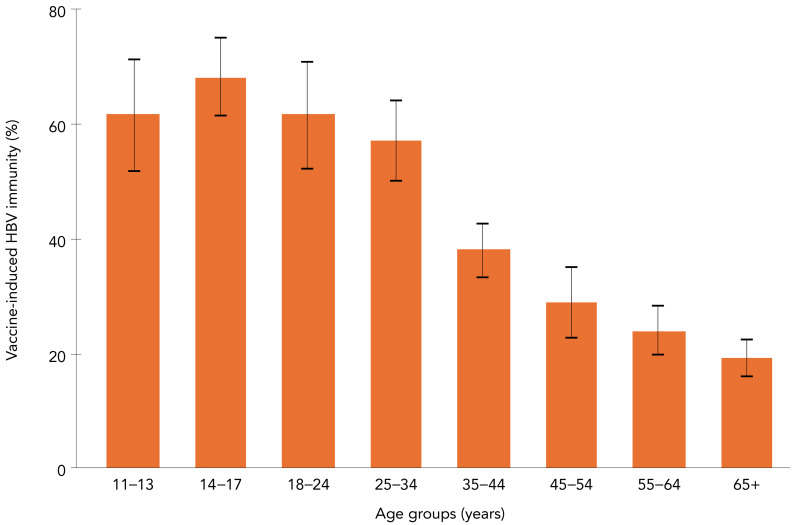
Vaccine-induced immunity to hepatitis B by age group, household population aged 11–79 years, Canada, 2016–2019

An estimated 39.0% (95% CI: 37.0%–41.0%) of the household population aged 11 to 79 years had laboratory evidence of vaccine-induced HBV immunity. There were statistically significant differences across age groups. Adolescents aged 14 to 17 years had the highest immunity at 68.3% (95% CI: 61.4%–75.2%), and adults aged 65 years or older had the lowest immunity at 19.4% (95% CI: 16.1%–22.7%). Immunity varied across regions, but differences did not reach statistical significance. People born outside of Canada had significantly lower vaccine-induced immunity (31.2%; 95% CI: 27.5%–35.0%) than those born in Canada (42.7%; 95% CI: 39.9%–45.4%; *p*<0.0001). Immunity was higher among people with higher household income, and those with higher level of education (**Table 5**, **Figure 1**). Among children and adolescents aged 11 to 17 years, 66.2% (95% CI: 62.1%–70.3%) had laboratory evidence of vaccine-induced immunity. Immunity varied by province or region (**Table 6**).

**Table 5:** Vaccine-induced immunity to hepatitis B, by selected characteristic, household population aged 11–79, Canada, 2016–2019 (n=7,541)

Characteristic	Weighted proportion (%)	95% CI	Weighted frequency (n)	95% CI	*p*
Overall	39.0	37.0–41.0	11,777,300	11,170,000–12,384,500	N/A
**Sex**					
Female	40.7	37.6–43.7	6,169,600	5,700,900–6,638,200	0.1
Male	37.4	34.6–40.2	5,607,700	5,185,800–6,029,600	
**Age groups**					
11–13	61.6	51.9–71.4	447,200	359,600–534,700	<0.0001
14–17	68.3	61.4–75.2	1,082,800	967,100–1,198,400	
18–24	61.7	52.3–71.0	1,828,000	1,424,300–2,231,600	
25–34	57.3	50.0–64.5	2,888,600	2,437,500–3,339,700	
35–44	38.1	33.1–43.1	1,900,500	1,495,000–2,306,000	
45–54	29.0	22.7–35.4	1,507,200	1,172,800–1,841,600	
55–64	24.1	19.7–28.5	1,248,800	992,900–1,504,600	
≥65	19.4	16.1–22.7	874,300	716,200–1,032,300	
**Province or region of residence**					
Atlantic	31.6	7.1–56.1	623,500	139,800–1,107,100	N/A
Québec	36.4	30.4–42.4	2,512,500	2,095,900–2,929,000	
Ontario	40.6	38.1–43.2	4,826,700	4,526,600–5,126,800	
Prairies	42.6	31.9–53.3	2,318,600	1,735,900–2,901,200	
British Columbia	37.4	27.5–47.4	1,496,100	1,097,200–1,895,000	
**Born outside of Canada**					
Yes	31.2	27.5–35.0	3,024,900	2,171,200–3,878,600	<0.0001
No	42.7	39.9–45.4	8,752,400	7,576,200–9,928,500	
**Household income**					
Lower	35.1	32.8–37.3	5,404,100	4,915,200–5,893,000	<0.0001
Higher	43.1	39.7–46.6	6,373,200	5,782,820–6,963,500	
**Education^a^**					
Less than secondary school graduation	20.2	14.2–26.2	491,600	308,300–675,000	<0.0001
Secondary school graduation	36.7	31.9–41.4	2,257,400	1,859,400–2,655,400	
Post-secondary graduation	39.2	36.8–41.7	7,465,900	6,953,500–7,978,200	

Abbreviations: CI, confidence interval; HBV, hepatitis B virus; N/A, not applicable

^a^ Among people aged >18 years (n=6,119)

**Table 6:** Vaccine-induced immunity to hepatitis B, by sex and region, household population aged 11–17, Canada, 2016–2019 (n=1,379)

Characteristic	Weighted proportion (%)	95% CI	Weighted frequency (n)	95% CI	*p*
Overall	66.2	62.1–70.3	1,529,900	1,391,900–1,668,000	N/A
**Sex**					
Female	64.6	59.5–69.7	763,300	685,100–841,600	0.4
Male	67.9	62.0–73.8	766,600	672,400–860,800	
**Province or region of residence**					
Atlantic	41.3	0.0–96.6	55,200	4,700–105,700	N/A
Québec	80.1	71.7–88.6	342,100	254,600–4,290,700	
Ontario	70.9	65.9–76	657,400	556,500–758,200	
Prairies	77.0	64.4–89.6	368,500	269,900–467,100	
British Columbia	31.0	4.5–57.4	106,700	22,000–191,400	

Abbreviations: CI, confidence interval; HBV, hepatitis B virus; N/A, not applicable

### Hepatitis C prevalence and awareness

The prevalence of past or present HCV infection (anti-HCV-positive) was 0.5% (95% CI: 0.2%–0.8%) in people aged 14 to 79 years. People born between 1945 and 1975 had the highest prevalence at 0.7% (95% CI: 0.2%–1.2%). Among people who reported ever injecting drugs, the prevalence was 9.3% (95% CI: 0.0%–19.5%). People living in households with lower income had significantly higher prevalence than those living in households with higher income, at 0.8% (95% CI: 0.3%–1.3%) compared to 0.2% (95% CI: 0.0%–0.4%; *p*=0.01) (**Table 7**).

**Table 7:** Estimated prevalence of past or present hepatitis C virus infection (antibody-positive prevalence) by selected characteristics, household population aged 14–79, Canada, 2016–2019 (n=7,044)

Characteristic	Weighted proportion (%)	95% CI	Weighted frequency (n)	95% CI	*p*
Overall	0.5	0.2–0.8	143,900	60,000–227,800	N/A
**Sex**					
Female	0.3	0.1–0.4	38,700	16,600–60,800	0.1
Male	0.7	0.1–1.3	105,200	19,600–190,800	
**Birth cohorts**					
<1945	-				0.2
1945–1975	0.7	0.2–1.2	102,500	33,500–171,500	
>1975	0.3	0.0–0.7	40,700	0–95,300	
**Lifetime history of injection drug use**					
Yes	9.3	0.0–19.5	29,700	8,300–51,000	0.2
No	0.4	0.1–0.7	113,700	28,829–198,700	
**Born outside of Canada**					
Yes	0.5	0.0–1.0	44,000	0–96,600	0.9
No	0.5	0.2–0.8	99,800	35,300–164,300	
**Born in an intermediate-to-high HCV prevalence country^a^**					
Yes	-				0.5
No	0.4	0.2–0.7	117,300	51,300–183,400	
**Household income**					
Lower	0.8	0.3–1.3	121,900	41,200–202,600	0.01
Higher	0.2	0.0–0.4	22,000	0–52,000	
**Education^b^**					
Less than secondary school graduation	2.1	0–4.6	51,200	0–109,400	0.3
Secondary school graduation	0.7	0–1.6	41,900	0–96,600	
Post-secondary graduation	0.3	0.1–0.4	50,800	0–82,600	

Abbreviations: CI, confidence interval; HCV, hepatitis C virus; N/A, not applicable; -, cell size does not meet diffusion guidelines

^a^ Countries with estimated hepatitis C antibodies prevalence greater or equal to 2%

^b^ Among people aged >18 years (n=6,121)

The prevalence of present HCV infection (RNA-positive) was 0.2% (95% CI: 0.0%–0.3%) among people aged 14 to 79 years. Among people born between 1945 and 1975, the prevalence was similar at 0.2% (95% CI: 0.0%–0.4%). Among people who reported ever injecting drugs, the prevalence was 5.7% (95% CI: 0.0%–13.5%) (**Table 8**). Of note, 37% of the anti-HCV-positive respondents were positive for RNA. Among people with present HCV infection, 51.2% (95% CI: 9.5%–92.9%) were aware of their infection. This proportion was 69.4% (95% CI: 13.2%–100.0%) among people born between 1945 and 1975 (**Table 9**).

**Table 8:** Estimated prevalence of present hepatitis C virus infection (RNA-positive prevalence) by selected characteristics, household population aged 14–79, Canada, 2016–2019 (n=7,044)

Characteristic	Weighted proportion (%)	95% CI	Weighted frequency (n)	95% CI	*p*
Overall	0.2	0.0–0.3	45,100	9,000–81,200	N/A
**Sex**					
Female	0.1	0.0–0.2	13,500	0–28,900	0.3
Male	0.2	0.0–0.5	31,600	0–65,700	
**Birth cohorts**					
<1945	-				0.8
1945–1975	0.2	0.0–0.4	31,000	0–65,100	
>1975	-				
**Lifetime history of injection drug use**					
Yes	5.7	0.0–13.5	18,000	180–35,900	0.4
No	0.1	0.0–0.2	27,100	0–59,700	
**Born outside of Canada**					
Yes	-				0.6
No	0.2	0.0–0.4	34,700	600–68,900	
**Born in an intermediate-to-high HCV prevalence country^a^**					
Yes	-				0.2
No	0.2	0.0–0.3	44,400	8,200–80,600	
**Household income**					
Lower	0.2	0.0–0.4	30,100	6,900–53,200	0.5
Higher	-				
**Education^b^**					
Secondary school graduation or less	0.2	0–0.3	31,300	0–65,100	1.0
Post-secondary graduation	0.2	0–0.4	13,800	0–30,700	

Abbreviations: CI, confidence interval; HCV, hepatitis C virus; N/A, not applicable; -, cell size does not meet diffusion guidelines

^a^ Countries with estimated hepatitis C antibodies prevalence greater or equal to 2%

^b^ Among people aged >18 years (n=6,121)

**Table 9:** Awareness of present hepatitis C virus infection, by selected characteristic, household population aged 14–79, Canada, 2016–2019 (n=14)

Characteristic	Weighted proportion (%)	95% CI	Weighted frequency (n)	95% CI	*p*
Overall	51.2	9.5–92.9	23,000	0–53,500	N/A
**Sex**					
Female	-				0.04
Male	69.4	24.3–100.0	22,000	0–52,500	
**Birth cohorts**					
<1945	-				N/A
1945–1975	63.4	13.2–100.0	19,600	0–49,900	
>1975	-				
**Born outside of Canada**					
Yes	-				0.2
No	64.4	21.1–100.0	22,400	0–53,000	
**Household income**					
Lower	26.8	0.0–64.6	8,000	0–16,300	N/A
Higher	-				
**Education**					
Secondary school graduation or less	-				0.3
Post-secondary graduation	63.9	11.1–100.0	20,000	0–50,200	

Abbreviations: CI, confidence interval; HCV, hepatitis C virus; N/A, not applicable; -, cell size does not meet diffusion guidelines

## Discussion

This study estimates the prevalence of HBV infection at 0.4% in the general population, of whom 49.0% were aware of their infection. The last estimates from the 2007–2009 and 2009–2011 cycles of the CHMS were 0.4% and 45.5% (95% CI: 21.3%–72.1%) for prevalence and proportion aware, respectively ((5)), indicating a relatively stable trend. The results of this study are comparable to a similar study done using the National Health and Nutrition Examination Survey (NHANES) data in the United States, which reported an HBsAg (and anti-HBc) prevalence of 0.2% (95% CI: 0.1%–0.3%) for the period 2017–March 2020 among people aged six years and older, among whom 49.8% (95% CI: 25.1%–74.6%) were aware of their infection ((11)). Another NHANES study for 2013–2018 estimated the HBsAg prevalence at 0.3% (95% CI: 0.2%–0.4%) for people aged six years or older, of which 65.6% were unaware ((12)).

This study estimates the prevalence of vaccine-induced HBV immunity at 39.0% (95% CI: 37.0%–41.0%) among 11- to 79-year-olds, and at 66.2% (95% CI: 62.1%–70.3%) among 11- to 17-year-olds, compared to “nearly 30%” of people aged 14 to 79 years reported by Roterman *et al.* ((5)), suggesting a small increase in immunity in recent years. The national vaccine coverage for 2019 (measured through self-reported or documented vaccine status) for at least one dose among adolescents aged 14 was much higher at 84.5% (82.1%–86.7%) ((13)), indicating an important difference between vaccination status, either documented or recalled, and laboratory evidence of immunity. One of the factors that could explain this difference is anti-HBs waning over time, therefore potentially leading to an underestimated vaccine-induced immunity. This could also contribute to explaining the differences in vaccine-immunity at the regional levels, given that the age at which HBV vaccines are offered varies from jurisdiction to jurisdiction (participants from provinces offering vaccination at birth or in infancy potentially showing waning over time and lower prevalence of anti-HBs, and those vaccinated later as part of school-based immunization potentially having higher prevalence of anti-HBs). Another factor that may explain the discrepancy between national vaccine coverage and our results is a potential overestimation of the vaccine coverage given that as it is measured for one dose or more, and the HBV-containing vaccines are routinely given as part of a two-dose or more vaccine schedule ((14)). A 2013–2018 NHANES study estimated that 21.4% (95% CI: 20.2%–22.6%) of people aged 25 years or more had vaccine-induced immunity (defined as anti-HBs positive). The study also found associations between younger age and being born in the United States and immunity ((12)).

This study estimates the prevalence of anti-HCV at 0.5%, and the prevalence of present HCV infection at 0.2%, of which 51.2% were aware of their infection. The last estimates from the CHMS were 0.5% for anti-HCV, of whom 30% were aware, indicating a stable trend with regard to past exposure ((5)). No RNA testing was performed in the previous study; therefore, awareness of current or past infection (anti-HCV-positive) cannot be compared to awareness of current infection (RNA-positive). Higher prevalence of present infection and proportion aware were reported for NHANES (2017–March 2020), respectively 0.9% (95% CI: 0.5%–1.4%) and 67.7% (50.2–82.2) ((15)). Another NHANES study for 2011–2016 identified a 2.3% viremic prevalence among people born between 1945 and 1965, a 0.47% viremic prevalence among foreign-born individuals, a viremic prevalence of 23.1% for PWID, and an inverse relationship between education level and prevalence, among others ((16)).

This study’s descriptive analysis has several strengths. First, representative, cross-sectional data of both diagnosed and undiagnosed individuals are a critical source of information for efforts to accurately depict the burden of disease, which have not been available since 2011. Second, RNA testing for HCV was added, which allows for a better characterization of the disease. Third, the weighting allows adjustment for survey non-response and allows for inference on the Canadian population. The distribution of sociodemographic variables, including age, sex, province or region, being born outside of Canada, and education for this study is comparable to their distribution in the population of Canada for 2021.

### Limitations

Several general limitations affect the findings. First, the CHMS excludes several groups of people and communities in Canada, which may affect generalizability of the findings. Second, the CHMS is likely to have underrepresentation of members of priority populations for STBBI (e.g., people who use drugs, or are incarcerated) who are likely to be disproportionately affected by HBV and HCV. Therefore, these results are considered to underestimate the “true” burden. In addition, several data elements of interest related to indigeneity, sexual orientation and sexual behaviours were not available. Third, some of this study’s results lack precision due to small sample sizes and should be interpreted and used with caution. Fourth, there is an increased risk of potential bias for regional estimates and subgroup analyses, as the survey was designed to be representative of the population of Canada as a whole. Fifth, immunity may have been underestimated, since anti-HBs wanes over time in vaccinated individuals, which is not necessarily indicative of a loss of protection ((14,17)). Lastly, the data source was insufficiently powered to address confounding and effect modification for the rare outcomes (prevalence of infections and awareness).

## Conclusion

This study provides updated estimates of HBV and HCV prevalence and the proportion of affected individuals who are aware of their infection, by sociodemographic characteristics. These results suggest a stable trend with regard to present HBV infection and awareness, as well as for exposure to hepatitis C in the household population aged 14 to 79. The results of this study should be used with caution where uncertainty is large and should be considered underestimates. Prevalence estimates from cross-sectional studies are essential to derive epidemiological estimates that include people who are unaware of their infection, in order to estimate the burden of disease. In addition, these analyses may be useful to inform future HBV and HCV screening guidance.
